# Policy makers, regulators and researchers’ perspectives on genomics research and the capacity of the National Health Research Act of 2013 to regulate genomics research in Zambia

**DOI:** 10.12688/aasopenres.13092.1

**Published:** 2020-07-24

**Authors:** Oliver Mweemba, John Musuku, Tulani Francis L. Matenga, Michael Parker, Rwamahe Rutakumwa, Janet Seeley, Twambo Simanga, Paulina Tindana, Jantina de Vries

**Affiliations:** 1 Department of Health Promotion and Education, School of Public Health, University of Zambia, Lusaka, Zambia, 10101, Zambia; 2 Children Hospital, University Teaching Hospitals, Lusaka, Zambia, 10101, Zambia; 3 Wellcome Centre for Ethics and Humanities (Ethox),Nuffield Department of Population Health, University of Oxford, Oxford, UK, UK; 4 Medical Research Council/Uganda Virus Research Institute and London School of Hygiene and Tropical Medicine, Uganda Research Unit, Entebbe, Uganda; 5 Department of Global Health and Development, London School of Hygiene and Tropical Medicine, London, UK; 6 Department of Health Policy, Planning and Management, School of Public Health College of Health Sciences University of Ghana, Accra, Ghana; 7 Department of Medicine, University of Cape Town, Cape Town, South Africa

**Keywords:** Zambia, genomics research, Health Research Act 2013, broad consent, sample sharing, data sharing, bio-banking

## Abstract

**Background:** Health research in sub-Saharan Africa takes place against a lengthy history of exploitation and unfair collaboration. This has involved the export of samples and data from the continent for the benefit of institutions and researchers elsewhere. In this paper, we report the perspectives of people involved in conducting genomics research in Zambia and the capacity of the Health Research Act (HRA) of 2013 in regulating genomics research.

**Methods**: We approached 14 purposively selected stakeholders involved in the development or implementation of the HRA in Zambia for in-depth interviews. These were members of research ethics committees, genomics researchers, Ministry of Health policy makers and institutional lawyers.

**Results**: Participants reported that there are benefits in genomics research for Zambia such as diagnosing and treatment of diseases. Participants also expressed concerns, most of which were ethical in nature. Prominent concerns were on consent. Participants’ main concern was the possible misuse of samples in the future. These concerns resonated with the HRA, which prohibits the use of broad consent for the collection of samples and data for future unspecified research. The implications of this is that Zambians may not participate in any kind of health research for which the storage, sharing and re-use of data or samples is envisaged. The restrictive nature of HRA means that genomics research may be excluded from future health research collaborations, thus isolating the country from potentially beneficial health research. Some policy makers also worried the samples and data that comes from such research may be difficult to access by local scientists.

**Conclusion**: In this article, we describe the views of Zambian policymakers on genomics research and the capacity of HRA in regulating genomics research. Our findings are relevant for the Zambian audience, and other African countries that are aiming to regulate health research, especially genomics research.

## Introduction

Health research in sub-Saharan Africa takes place against a lengthy history of exploitation and unfair collaboration. Characterizing this trend is a history of export of samples and data from the continent for the benefit of institutions and researchers elsewhere ( Heymann *et al*., 2016; Munung *et al.*, 2017). While the H3Africa initiative aims to establish biobanks in Africa, much other genomics research, where the export of samples and data for use elsewhere, continues to be common, partly because of mandatory policies for the sharing of samples and data. Considering such trends, genomics research is seen as perpetuating biocolonialism ( Van Rinsum & Godfrey, 2004)

As the birthplace of modern humans and the home of the greatest human genomic diversity, the African continent is a logical focus for efforts aimed at improving diversity, inclusion, and representation in genomics research ( Bentley *et al*., 2019). Meanwhile, African policymakers involved in the regulation of health research struggle to make sense of the need to, on the one hand, promote health research and collaboration, but on the other hand protect researchers and the general public from (perceived and/or real) exploitation. A particular challenge in this regard is that the human capacity and infrastructure required to successfully conduct genomics research remains concentrated in particular African countries ( Aron *et al*., 2017), with others relying on collaboration to make this happen ( Yakubu *et al*., 2018).

Several authors have described how different regulatory regimes across the continent strike a balance between these apparently opposing tensions ( Barchi & Little, 2016; Nienaber, 2011; Staunton & Moodley, 2013). Whilst many African countries have not effectively regulated the export of samples and data, others have created regulatory frameworks that are progressive – for instance, Rwanda, Ethiopia, Uganda, and Nigeria have introduced regulation that strikes a balance between promoting research whilst protecting national interests ( de Vries *et al*., 2017). The picture that emerges from such analyses is that, overall, African countries tend to be permissive towards genomics research and its associated policies regarding the storage, redistribution, and re-use of samples and data for future unspecified research. They are also actively or passively permissive towards the use of broader forms of consent to allow the re-use of samples and data ( Tindana & de Vries, 2016).

A notable exception is Zambia, which has specifically regulated against key policy components of health research. In 2013, the Health Research Act No 2 of 2013 of the Laws of Zambia (henceforth the HRA 2013) came into effect. As is evident from the name, this law specifically regulates health research and is one of the few specific health research acts on the continent. Although the HRA 2013 is a comprehensive law that aims to afford protections for the Zambian population, there are two components of this Act that challenge the conduct of genomics research in Zambia ( Chanda-Kapata *et al*., 2015). The first is a requirement that no biological materials may be collected for ‘future unspecified health research’ (HRA Section 47(1)) – effectively prohibiting the use of blanket, broad or tiered consent (although exactly what counts as ‘unspecified' could be debated). The second relates to tight regulations around the storage, export, and re-use of tissue samples. For instance, biological materials may not be stored for ‘unspecified storage' (HRA Section 47(1)) and may only be exported under strict conditions.

The overall gist of the legislation seems to be to promote Zambian domestic research capacity, including Zambian’s ability to store, process and use samples and data. Yet in a commentary on the HRA, Chanda-Kapata *et al.* (2015) articulated a concern that these provisions could have a dampening effect on genomics research in Zambia. Anecdotally, we know of instances where the strict regulatory environment has already impacted on genomics research conducted in the country, with Zambians being excluded from participating in international health genomics research that could have generated findings relevant to Zambians’ health. Against this background, we explored the perspectives of policy makers and regulators on the benefits of genomics research, as well as the concerns and the capacity of the HRA in conducting genomics research in Zambia. Such research is important in order to provide an opportunity for research stakeholders, e.g. policy makers, researchers, ethics committee members, to share their reflections on advances in genomics research and the implications for (and of) the existing laws and regulations.

## Methods

To investigate our study aim, we employed a qualitative case study approach where we approached a range of stakeholders involved in genomics research and in the development or implementation of the HRA in Zambia for in-depth interviews. Specifically, we invited members of research ethics committees, researchers in genomics, Ministry of Health policy makers and an institutional lawyer to participate in this study.

### Study site

The study was conducted by the Department of Health Promotion and Education at the University of Zambia and the University of Cape Town. We interviewed participants from selected institutions in Zambia in their various locations.

### Study sample

The study reported here was part of a larger study in which we also interviewed participants in a rheumatic heart disease genomics study, reported in Mweemba *et al.* (2019). Here, we report on findings resulting from in-depth interviews with people involved in the regulation or implementation of research policies for genomics research and generally health research in Zambia.

We approached 16 participants to participate in the study, but only interviewed 14 participants. The other two, despite accepting to participate, were constantly busy and mostly out of the country. Among the 14 we interviewed, some had dual roles: 7 members of institutional Research Ethics Committees (5 of them were also health researchers with experience in conducting and reviewing genomics research); 5 members of the Zambian National Health Research Ethics Committee (NHREC) (3 of whom also served on an institutional committee); 2 scientists involved in genomics research; 1 member of the Ministry of Health involved in the development of the HRA; 1 community representative; and 1 institutional lawyer. We initially identified participants using our own network and knowledge of the Zambian environment and then asked key interviewees for suggestions as to who else to speak with (snowball sampling). While each interview contributed unique perspectives on the topics discussed, we started reaching saturation on most topics by the 10th interview. Nevertheless, we continued with four more not only to increase diversity of participants but also to ensure that we reached saturation on all the key issues in the study.

Interested participants were contacted by phone to explain the study and schedule an appointment. Specifically, in the interviews, we explored participants’ views on genomics research and broad consent in the context of health research and the history and capacity of the Zambian Health Research Act of 2013 to regulate genomics in Zambia. Interviews were conducted between June and December 2017. The specific topics discussed included perspectives on genomics and bio-banking research, data and sample sharing, consent models for genomics research, the health research Act of 2013, and international collaborations and governance issues (see *Extended data:* interview guides ( Mweemba *et al*., 2020)). The first author (OM) interviewed all the participants. He has a PhD in Social Science and Health and has extensive experience in qualitative interviewing on complex health research topics. At the time of the study, he was a Lecturer in the Department of Health Promotion and Education at the School of Public Health, University of Zambia. OM had well-established relationship with all the participants as fellow researchers and colleagues dating many years to 2012. The typical duration of the interviews was about 30 minutes. All interviews were conducted privately in the respective offices of the different participants.

### Data analysis

All interviews were audio-recorded and notes written during the interviews. All interviews were transcribed verbatim. Transcripts were not returned to participants for checking. Following transcription, text files were imported into NVivo 11 (QSR International Pty Ltd, 2015) as part of the larger dataset including interviews not analysed for this paper (see Mweemba *et al*., 2019). Two researchers (OM and JdV) independently coded five transcripts, including two transcripts from the particular dataset analysed for this paper. Following discussions, we developed a hierarchical coding scheme together with a codebook (see *Extended* data ( Mweemba *et al*., 2020)) describing each code and its relationship to overarching study themes. Interviews were then coded by the researchers and two research assistants. Two researchers (OM and JdV) together analysed and interpreted coded data using the Framework Method functionality embedded in NVivo 11 ( Gale *et al*., 2013; Smith & Firth, 2011). We discussed data interpretations with the wider study team.

### Ethics approval and consent

All the participants provided written consent after explaining the purpose of the study, including the possible risks and benefits of the study. A signed copy of each set of consent documents were kept by the study participants. We obtained ethics approval for this study from the University of Cape Town’s Faculty of Health Sciences Health Research Ethics Committee (approval number FHS644-2015) and the University of Zambia Biomedical Research Ethics Committee (approval number 001-09-2016).

## Results

The results below are presented in three inter-related broader themes: the benefits of genomics research in Zambia; concerns about genomics research; and the Zambia National Health Research Act and regulation of genomics research in Zambia.

### The benefits of genomics research in Zambia

The participants largely understood genomics research in the context of health research as a scientific approach to determining the genetic causes of diseases. Some participants also indicated that genomics research could help develop and target health policies and devise health promotion support programs for individuals and groups with the potential to be affected by a particular disease or health condition. Overall, participants seemed to agree that there is value to promoting genomics research in Zambia because of its likely benefit for the Zambian population.

*It’s a very important area in that you’re trying to find out the genetic determinants of disease you know […] in Africa I think it’s an area that hasn’t been very well explored … but it could generate quite useful information that can help us in disease prevention, disease treatment, management of patients with particular conditions… I believe that we cannot do without genomics research, it is extremely important (Researcher and former Research Ethics Committee member*).

Participants also suggested that Zambian researchers should be supported to participate in international genomics research collaborations.

*If we are to understand disease dynamics, sometimes we need to do those studies. If we are to develop treatments for certain diseases, we need to know how these drugs operate in the human body, what changes happen in the chromosomes and so on… For clinical practice, if I could relate to drugs that is what I understand better yes probably because that could help us now individualise the management of patients. So as a medical person I think we need to encourage genomics research. (Researcher and Research Ethics Committee member*).

### Concerns about genomics research

Whilst recognizing its potential benefits, the participants also expressed concerns about genomics research and most of these were of an ethical nature. The issue of consent was raised frequently. Although less controversial than blanket consent, participants were concerned about consent for future unspecified studies (be that through broad or tiered consent) with the main concern being the possible misuse of samples in the future.

*Those are some of the issues that come up when you are talking about genomics research and it creates a problem […] you can’t get consent for every single thing you are going to do in future from the patient but if there is a mechanism to perhaps get consent from the ethics committee for each new type of study that might be helpful because then you have a situation where a researcher wants to go overboard and to do things wrong. But that is still not enough because some people might still do wrong things anyway (Research Ethics Committee member).*

Interestingly, participants framed informed consent as being one way of protecting against sample misuse:

*There is a likelihood that things that the person has not consented to are going to be done and of course we have had this issue that has been discussed left right and centre of how people from the western world, for example, want to manipulate Africans by coming here and harvest samples and things like that. Many people are in a way duped so to say because they may be enticed with something and they give these samples (Institutional Lawyer).*

Issues around consent were also framed in terms of an ‘attachment’ of participants to their samples. The risk is that participants could lose trust in the research system if samples were used indiscriminately.

*I think the issues of people have this attachment to their samples, their blood and most of their concerns lie from say maybe no trust or lack of trust based on maybe unknown things or stigma and so some of the ethical issues that come up will be if there is no disclosure what would the patient think if they found out later on that their blood has been used for a specific type of research which they did not approve or they find offensive…maybe offensive to their culture or their religious beliefs (Research Ethics Committee member).*

Because of some of the concerns raised, specific consent was insisted upon as a possible solution to these concerns. Specific consent is defined as consent for a particular research question as opposed to for instance tiered or broad consent, which allow for the re-use of samples and data for other research questions also.

*We have to specify what exactly the individual is consenting to, what can be done on that particular specimen they are submitting or they are donating and specifically that they would like the specimen to be used for the particular condition the participant consented for (Former Research Ethics Committee and NHREC member)*.
*Let us be specific and let us predict a bit what we are thinking so that you don’t just hear people saying no we will put it in the bio-bank until 2021, what you are going to do in 2021. Just state and discuss things so that people know where you are going and what you are going to do (Research Ethics Committee and NHREC member).*

Participants also framed concerns over sample sharing and export in terms of sample misuse and specifically the potential for samples and data to be used in ways that reflect badly on Zambia, or on particular communities.

*[In one genomics study we reviewed], I think that was the one where the samples were going to be shipped off to somewhere […] Some people feel that by doing that you are surrendering ownership to somebody else and of course they felt that maybe some of the research will not be in good light in terms of people trying to find a way of dealing [harm] with you, by looking at the genetic make-up (Researcher and former Research Ethics Committee member).*

In this quote, ‘dealing with you’ is a commonly used reference to the historical colonization of Zambia.

Many of the participants placed emphasis on the importance of *control* and *ownership* by Zambian researchers and regulatory institutions over samples and data, which could leave samples open to abuse by unscrupulous researchers.

*Earlier there was a suspicion that was associated with genomics research that, “Why are people getting these samples, sending them abroad for analysis and so on, what else are they going to do there because once the samples leave the country; we have no control on what happens… […] we are not able to regulate [whether] they adhere to what they have said they are going to do out there” (Research Ethics Committee member).*

Such concerns were articulated not just in relation to the export of samples but also to the sharing of genomic data. Important in the context of the latter were concerns about benefits, sometimes articulated as intellectual property rights.

*This information leaves the country and we have no say. That’s a security issue but also just intellectual property, what happens to the information that you generated from this? Do we get any benefits from it, can we use it to better the lives of our people? (Senior Government Official and Policy Maker).*

Building on a broader question of benefits and benefit sharing, participants questioned whether samples and data would likely still be available to Zambian researchers or students for further research. Past experience is that it is difficult for local researchers to access samples and data from Zambia that is stored and distributed from other countries, tapping into broader concerns about exploitation and fairness in collaboration.

*We have had situations where data that is produced, here in Zambia, people are having problems accessing it. But when you go to the North you find the same data […] easily accessible […]. Another experience is… you have this data set which you enter online but once it gets into the database you can’t access the whole database. The database server is in [somewhere in the North]… they have the monopoly on the data and the only thing you can do is create the data which you can’t access (Researcher, Research Ethics Committee member and former NHREC member)*

### The National Health Research Act and regulation of genomics research

Amidst these concerns, the participants commented on the appropriateness and adequacy of the National Health Research Act of 2013 in regulating genomics research in Zambia. Importantly, out of the 14 interviewees – all of whom were involved either with the development of the Act or are charged with its implementation as members of ethics committees – about eight participants were conversant with the law and the rest had general ideas about it. All the eight participants who were conversant with the law were involved either with the development of the Act or its implementation.

The Act was drafted following an increasing number of clinical trials and other health research projects being conducted in Zambia, and the realisation by key stakeholders (some of whom we interviewed) that the existing regulations were not adequate to regulate such research. A specific concern at the time related to the export of samples for further analysis to other countries that had stronger laboratory capacity.

*There was this rampant exportation of samples without regulation it was therefore important that there had to be regulation in terms of biological materials. Secondly […] as the National Health Ethics Committee was developed, it recognised that there was no legal framework in which the ethics would be empowered to follow-up studies, hence the need formulate the Act. So, it was discovered that actually in Zambia at that particular time there was generally no law which allowed for research on human beings. So, there was a time that research in live human beings was suspended until we came up with this (Researcher and former NHREC member).*

Participants described that the Act had been important in regulating the export of samples with specific agreements on how the samples will be used detailed in the now-mandatory Material Transfer Agreement.

*There was a suspicion that was associated with genomics research that why are people getting these samples, sending them abroad for analysis, what else are they going to do […] So I think that was the major concern by the committee at the time but it has since reduced because I think the modalities of exporting samples now are becoming a bit stringent yes with the coming of the material transfer agreement. […] I think samples were just going out without any regulations but now they are being regulated (Research Ethics Committee member).*

Yet participants also described that once enacted, the new Act brought about considerable debate and controversy in Zambia, particularly about: the ownership of data and samples by the government; approval of publications by the government; and tightening regulations about the establishment of biobanks.

*I think there's a statement which says that "specimens belong to the Government of the Republic of Zambia" which in essence actually says government should have jurisdiction on what happens to specimens once they have been collected and then secondly there is I think there is a part which says that before any publication, before you can publish data arising from a particular research, there should be authorisation by the Ministry of Health […]. That was seen as if it is stifling research and publications (Researcher and former Research Ethics Committee member).*

Another point of debate related to the prohibition to collect specimens for ‘unspecified health research’. Our research participants described that some provisions of the Act were at odds with genomics research policies, including the use of consent for future use of samples and data which is not allowed in the Act. Part of the reason for these restrictive policies was that at the time of its development, knowledge on genomics research was not common, which explains why some restrictions were at odds with the requirements of such research.

*Probably at the beginning when these guidelines and these provisions were being drafted, there was no clear understanding of what genomic research was all about because for example if you say you are not going to allow submission or transportation of samples to a particular biobank even if it’s for genetic research then in essence what you are saying is each particular country should hold their samples, you know but I mean there are some conditions which are cross-cutting and […] common in a particular region and I don’t think its cost-effective to have biobanks in each country (Researcher and former NHREC member).*

Participants also highlighted that because the Act had no jurisdiction beyond the borders, this made it difficult to monitor and regulate the use of samples once they leave the country, including ensuring that the researchers adhere to terms of the agreement on the use of samples. Importantly, the participants acknowledged that the stringency of the Act may preclude Zambians from effectively engaging in genomics research.

*I think that discussions [to consider changes] on that started after the Livingstone meeting ^1^ […] that's when well a lot of people realized that our colleagues in the region have been engaged in genomic research for some time and we believe that there is a lot of good that can come out of that particular research and at the same time we understand that and believe that we are part of the global village; conditions that affect our people here that we feel that the solutions, could be partly found from genomic research but then given, the stringency of our regulations it was going to be very difficult for us as a country to participate in genomic research (Researcher and former Research Ethics Committee member).*

Yet against such concerns, participants felt it was really important to ensure that Zambian researchers are protected and that there are provisions in place that would ensure the long-term reversal of a trend of sample extraction without benefits to Zambian researchers or the general public.

*You cannot perpetually be transferring materials we need to make sure that the Zambian institutions are also empowered. It means that if we know that in future studies we are going to need this, it is better to make arrangements that that equipment is brought in the country and then for future studies we don’t have to ship materials outside (Institutional and NHREC member).*

One way in which the Act protects Zambian investigators is by requiring that international research collaborations involve a Zambian investigator based in a local institution.

*As much as the Act is limited it at least touches on all these things, one of the things that it talks about the collaboration that whenever there is a study going on in the country there should at least be a Zambian either the PI or the Co-PI should be a local individual who is placed here who can be traced to actually have research contacts in the country.... (Senior Government Official and Policy Maker).*

Generally, the policymakers we interviewed acknowledged that the Act in its ‘current’ form was limited in its ability to provide adequate regulation of genomics research. At the same time, however, some policymakers acknowledged that it would take a long time to amend the law. In view of this, some policymakers were optimistic that genomics research can be approved despite the legal circumstances if a convincing case is made by researchers to the ethics committee, as was the case with the Rheumatic Heart Disease Genomics study conducted at the University of Zambia (see for instance Mweemba *et al*., 2019).

*There have been some discussions which say that they are [moving] towards amending the law but you know how long it takes to even just enact a law later on, it requires political will so our hope is that once the authority, because the law provides for the national health research authority to be put in place, once that is put in place and starts running the people within that authority will be able to move the agenda… in a case where researchers put their case convincingly to the ethics committee, the reasons are ok, the study is justified. I don't see the ethics committee refusing to do this kind of study… I believe that the ethics committee can do that if they know what they are doing by making sure that information is anonymous cannot be traced back to the individuals and if it comes out, it comes out in such a way that doesn't stigmatise the whole community (Former NHREC member).*

Interestingly, participants described how they sought to navigate this prohibition by seeking to get some clarity – at a broad level – of what future research could be conducted.

*[The law] says that samples shall not be collected for unspecified future studies and so we have problems to reconcile that with the need to facilitate research but what we would advise people would be to try to be as specific as they could. You know you cannot predict what you are going to do in future but if you can go some way towards specifying then, we would approve. But if it’s completely blank like unspecified future studies then we would be going against the spirit of the law and we would not approve. That is not to say that the law is ok as it is, it might need to be reviewed but because it is currently the law you have to still meet the requirements (Former NHREC member and serving member of an Institutional Ethics Committee).*

The participants recommended that what was needed is to strengthen the regulatory system overall and specifically for genomics research. They suggested that new guidelines should be developed specifically aiming to address the issues that arise in genomics research including broad consent, sample and data sharing, sample storage and the feedback of results.

*Well, probably the best approach would be to have national guidelines on genomic research […] so that when we are for instance reviewing genomics research we know that these are the guidelines that should apply (Research Ethics Committee member).*

Importantly, it was also argued that guidelines governing research collaborations ought to be developed which detail issues such as data and sample ownership, agreements about publications and authorship and so forth. These regulatory frameworks should include mechanisms for regulating the samples that leave the country including biobanks that are situated in other countries and that contain samples from Zambia.

## Discussion

The empirical data generated by our study reflects participants’ perspectives on genomics research as well as the concerns and the effect and capacity of the NHA to regulate genomics research. Although participants agreed that there is value in promoting genomics research in Zambia, they also expressed concern over key ethical issues that such research raises. Primary amongst these was a worry about the potential for genomic resources to be misused in ways that offend the culture or religion of research participants, or that may be stigmatising or exploitative in nature. Importantly, such concerns suggested the need to manage tension between the need for control and protection on the one hand and permissive policies that allowed to facilitate collaboration. In an under-resourced country like Zambia where there are no large-scale sequencing facilities available, the export of samples for genotyping or sequencing may be the only way to ensure the inclusion of Zambian research participants in genomics research. An alternative would be large-scale and long-term government investment in genomic research infrastructure.

Perhaps the reason our participants were concerned about sample export can be explained by the emphasis they placed on the importance of oversight and ownership by Zambian researchers and institutions over samples and data. They described that a problematic feature of sample export is that Zambian regulatory institutions lose ‘control’ and that this could leave samples open to abuse by unscrupulous researchers. Importantly, however, participants struggled to give examples of what would constitute abuse. Our findings align with those by Tindana *et al.* (2014) who also revealed that given the inevitable uncertainty of future uses, perceptions of unfairness in who gains from sample export and storage, and fears of losing control of samples after export were expressed by their participants in a study conducted in Ghana and Kenya.

Due to the nature of genomics research, it involves the establishment of large and diverse scientific networks bringing together diverse and interdependent forms of expertise and institutions in higher and lower-income countries. Participants were concerned about the lack of capacity of local researchers to meaningfully participate in international collaborative genomics research. Out of concern over historic exploitation and perpetuation of inequalities by sending African samples to non-African laboratories ( Matenga *et al*., 2019; Okeke, 2016), some African nations have strict guidelines regulating the export of samples ( de Vries *et al*., 2017). Petti *et al.* (2006) suggest that performing laboratory tests locally or at least regionally gives greater African ownership of studies and high income countries collaborators need to help build research capacity.

Regarding the Health Research Act No 2 of 2013 of the Laws of Zambia, although a comprehensive law, the prohibitive provisions on the use of broad consent for the collection of samples and data for future unspecified research and the tight regulations around the storage, export, and re-use of tissue samples means that Zambians are currently restricted in their involvement in any kind of health research for which storage, sharing and re-use of data or samples is envisaged. Yet sharing, storage and re-use of samples and data are an increasingly common component of health research globally, including in clinical trials research, genomics, and research on emerging drug resistance for instance. A worry is that the strict regulatory environment in Zambia could mean that the country and its researchers are avoided in future health research collaborations, thus isolating the country from potentially beneficial health research.

Interestingly, our participants did not seem to share this concern and generally supported this provision of the Act. The consent and export restrictions of the Act were conceptualised as important ways of protecting Zambian research participants and researchers from exploitation by researchers and institutions in other countries – and mainly in high-income countries. They were also seen as appropriate means to protect individuals against research that could lead to stigma or that could otherwise be seen as offensive from a cultural or religious perspective. This narrative is not unique to Zambia. It also been reported in other studies from other countries ( Moodley *et al*., 2014; Igbe & Adebamowo, 2012). This suggest that the legacy of exploitive research in Africa still lingers in the minds of regulators and policy makers.

Our study suggests that the legislation is arguably not the best way to guard against perceived and actual unfair research practices, especially in the field of health research that is constantly changing. Regulation that has the ability to adapt to these changes may be better suited for that purpose. But beyond that question, what stands out is that health research in Zambia takes place against a context of deep mistrust in the intentions and integrity of international researchers. The open science policies on which genomics is premised – involving sharing, storage and future use of samples and data – need to take account of this reality.

## Conclusion

The results of this research suggest that although most of the stakeholders we interviewed agreed that conducting genomics research in Zambia could be beneficial, some concerns were expressed around the nature of consent for these types of studies and the potential for misuse of samples in future. Though these concerns resonated with the HRA, which prohibits the use of consent for the collection of samples and data for future unspecified research, some participants indicated that the HRA in its ‘current’ form was limited in its capacity to regulate genomics and bio-banking research in Zambia.

Our findings are relevant not just for the Zambian audience, but also for those in other African countries that are aiming to regulate health research especially genomics research. Specifically, we hope that this paper has provided some aspects that need to be considered in national regulation of genomics research including control over samples and data. We also hope future international collaboration in genomics research will consider the concerns that arise from this study.

## Data availability

Due to the small number of interview participants who also hold strategic positions in Universities, Governments and in the community, sharing their information will compromise their identity and raise confidentiality and privacy concerns for the participants, and hence jeopardise their position or their role in their organisations. We have provided information in form of quotes, but these are highly anonymised to protect the participants' identities. The University of Zambia Biomedical Ethics Committee that approved this study, approved it under the strict terms of maintaining privacy and confidentiality and their default position for ethical approval does not include data sharing unless its specifically requested for and justifications for doing should be provided. Unfortunately, for these interviews, data sharing was not requested for because of the nature of interviews, which identifies the participant's role in their organisation and their identities, as stated above. Those interested may contact us for any clarifications on the research data, and we can share only broader and processed information to protect the identity of individual participants and their institutions, such as matrix tables, summaries of themes and further quotes on specific topics. We will also be happy to share the data collection tools used in the study and the code book we generated for this paper (see *Extended data* ( Mweemba *et al*., 2020)).

### Extended data

Figshare: Extended data for the Genomics Study in Zambia, http://www.doi.org/10.6084/m9.figshare.12639200.v1 ( Mweemba *et al*., 2020).

- Interview Guide Research Regulators V2
- Interview Guide Research Ethics Committee Members/Researchers V2
- Codebook

### Reporting guidelines

Figshare: COREQ checklist for ‘Policy makers, regulators and researchers’ perspectives on genomics research and the capacity of the National Health Research Act of 2013 to regulate genomics research in Zambia’, http://www.doi.org/10.6084/m9.figshare.12639200.v1 ( Mweemba *et al*., 2020).

Data are available under the terms of the Creative Commons Zero "No rights reserved" data waiver (CC0 1.0 Public domain dedication).

## Footnotes

^1^ The H3Africa Consortium organized a series of engagement meetings with members of ethics committees and policymakers from across the continent. For a description of the Livingstone meeting, see De Vries J, Littler K, Matimba A, McCurdy S, Ouwe Missi Oukem-Boyer O, Seeley J, Tindana P: Evolving perspectives on broad consent for genomics research and biobanking in Africa. Report of the Second H3Africa Ethics Consultation Meeting, 11th May 2015. Global Health, Epidemiology and Genomics 2016, 1(e13):1-3.
