# Supplementary material for: Policy makers, regulators and researchers’ perspectives on genomics research and the capacity of the National Health Research Act of 2013 to regulate genomics research in Zambia
Source: AAS Open Res. 2024 Dec 2;3:32. Originally published 2020 Jul 24. [Version 2] doi: 10.12688/aasopenres.13092.2 (PMC12770881; doi:10.12688/aasopenres.13092.2)
Supplement: Peer review history

# Reviewer report: Reviewer response for version 2

**DOI:** 10.21956/aasopenres.16779.r33457

**Published:** 2026-02-04

**Authors:** Emma Griffiths, Charlotte Barclay

**Affiliations:** 1 Simon Fraser University, Burnaby, Canada; 2 Faculty of Health Sciences, Simon Fraser University Faculty of Health Sciences (Ringgold ID: 241829), Vancouver, British Columbia, Canada

The manuscript “Policy makers, regulators and researchers’ perspectives on genomics research and the capacity of the National Health Research Act of 2013 to regulate research in Zambia” aims to understand where regulatory boundaries should be set with regards to genomics research so that innovation in Zambia is not stifled but risks of exploitation and opportunity loss are appropriately managed. The paper describes the results of a survey conducted with 14 participants involved in research ethics committees, genomics research, policy making, and institutional legal matters. A structured questionnaire was prepared, and interviews were performed. Transcribed interview responses were anonymized and coded, and summarized in the manuscript with selected quotes as support for general findings. 

 The work captures concerns about genomics research and the regulatory powers of Zambia’s National Research Health Act (2013). Such discussions are of much interest especially in light of international PABS frameworks under consideration, and global negotiations about the sharing of materials and data.

 I have some concerns about some critical limitations of the study that have not been explicitly stated, and some suggestions. 

 -The study only involves 14 participants. The sample size is extremely small, and this limits what data can be shared. For example, we are given selected quotes but can’t see the responses to all the questions because of privacy concerns. We have to trust that the authors are representing the responses appropriately but cannot assess this ourselves. Also, statistical analyses cannot be performed, so we are not sure whether the quotes represent the general feelings of the participants, or only one person. We are also not given much information about the individual’s experience with genomics. Because the sample size is so small, the results could be very easily biased towards individuals with many positive or negative experiences - or individuals with more theoretical experience vs practical real-world implementation and health decision-making. Furthermore, many of the quotes are somewhat general and at times vague, and do not cite specific examples from their experience. Without knowing the individuals’ experiences, it is hard to evaluate their feedback, and these details are difficult to provide for confidentiality reasons. With a larger sample size, these issues would likely introduce less bias.   

 - The authors state in the introduction that genetic and genomic research requires access to human DNA. This is only true for human genomics research, not for pathogen genomics research, nor genomics research on other organisms (e.g. plants/animals). The entire study is geared toward human genomics research but the distinction between human and other research (e.g. pathogen) is not made. The participants were not asked if that distinction makes a difference in how samples and data should be regulated. Samples collected for public health pathogen surveillance, for example, often do not require consent. Data is shared publicly for national and international situational awareness and subsequently may be used for secondary analysis. How is this addressed by the NRHA2013 and the study? The authors should clarify that their study only involved regulatory considerations of human genomics research.

 - The questionnaire seems leading in some places, with the interviewers immediately jumping from questions about awareness of phenomena to concerns e.g. in sections about Data & Sample Sharing, Consent Modules, Perspectives in Genomics & Biobanking, the questions go straight from “Do you know about ….” to “What are your concerns”. This presupposes that the interviewee already has concerns with these subjects.

 - Interviews took place in 2017, and it is now 2026 - almost a decade later and post a global pandemic where genomic data sharing was vital in identifying new variants of concern, tracing transmission routes, and identifying sources of infection. It would be interesting to know if anything has changed in that time, and whether NRHA2013 is under/has undergone review.

 -It would be helpful for the reader if the authors could summarize the key points of NRHA2013 that pertain to genomics research so the reader can better understand the extent of its powers. 

 The work provides an interesting starting point for further study, and the authors have clearly invested a lot of effort in collecting data and analysis. The manuscript is well written, and is clear and concise. The authors have provided their survey instruments and coding dictionary openly, which is great. Taken together, I would recommend 1) that the authors acknowledge the issues of the small sample size in the Limitations section, 2) clarify that the study was about human genomics research (or change the definition in the introduction), 3) provide a summary table highlighting key regulatory points of NRHA2013, and 4) provide information as to updates to the Act. If these concerns are addressed, I would recommend the study for Indexing.

Is the work clearly and accurately presented and does it cite the current literature?

Yes

If applicable, is the statistical analysis and its interpretation appropriate?

Not applicable

Are all the source data underlying the results available to ensure full reproducibility?

Partly

Is the study design appropriate and is the work technically sound?

Partly

Are the conclusions drawn adequately supported by the results?

Yes

Are sufficient details of methods and analysis provided to allow replication by others?

Partly

Reviewer Expertise:

pathogen genomics, public health, data standards development, data interoperability, needs assessment

We confirm that we have read this submission and believe that we have an appropriate level of expertise to confirm that it is of an acceptable scientific standard, however we have significant reservations, as outlined above.

# Reviewer report: Reviewer response for version 2

**DOI:** 10.21956/aasopenres.16779.r33070

**Published:** 2026-01-05

**Authors:** Albert Vernon Smith

**Affiliations:** 1 University of Michigan, Ann Arbor, michigan, USA

This is an interesting study examining the regulation of genomics research in Zambia via a statue enacted in 2013. In reading the article, I note many excellent questions already raised in peer review, which have largely been addressed by the authors in an updated version. 

 As a researcher outside Zambia and Africa, some of my immediate thoughts relate to how the policy may have directly affected participation and collaboration with international genomic studies. While outside the scope of this article, has the Act affected ability of Zambian researchers to participate in international collaborations? Similarly, has it limited potential investment and development in the genomics sector? The article frames the concerns couched in ways such as "potentially restrict" and "genomics research may be excluded". With the survey design, it is not possible to address this issue in any statistically meaningful way. The article does acknowledge unspecified cases of inability to participate. It would be good to cite specific examples (though this likely is not possible for understandable reasons). In this regard, it would be nice to understand the perspective of researchers outside Zambia with views on their ability to collaborate with and support genomic research in Zambia. 

 As another general thought, it would be good to add additional perspective on how thinking might have evolved. While the interviewed participants seem largely supportive of the Act, perhaps there could be additional reflection on updates that could balance proper data usage with effective participation in contemporary collaborative cross-national genomic studies. Thoughts that come to mind how consent tied to IRB review might be able to safeguard participants interests. Also, could a model where subjects are reconsented make regulators and participants more open to broad or tiered consent? Similarly, would an ability to withdraw participation affect attitudes? 

 If it exists, is there any evidence that the Act has increased participation by subjects in Zambian genomic studies (because individuals feel their interests are better protected by the Act)? 

 On the specific questions above.

 --

 Are sufficient details of methods and analysis provided to allow replication by others?

 --

 Given privacy concerns the authors are not able to provide full transcripts of answers. Therefore, it is not possible to evaluate how fully the highlighted quotes reflect the broad conclusions. 

 The authors raise a concept of "saturation". It would be good to understand how that could be defined in a meaningful way. Given that a majority of the participants serve on an ethics committee there could be some limitations in the diversity of thinking. Where are the places were there are difference of opinion? They must exist. It would have good to have more than two perspectives from "genomics researchers". There is potential for greater diversity of opinion from a regulatory perspective. Phrased as question, are there any differences of opinion based on the roles of participants (as cited: ethics committee members, genomics researchers, policy maker, community participant, and lawyer).

 A closing question in the survey asks for "recommendations for addressing challenges". This is open ended phrasing, and it be good to have insight on specific recommendations and possible changes. It would be good if specific questions would have been asked to survey participants.

 --

 If applicable, is the statistical analysis and its interpretation appropriate?

 --

 This is not applicable, as the study isn't designed for statistical analysis.

 --

 Are all the source data underlying the results available to ensure full reproducibility?

 --

 As noted in a previous comment on methods, this is not possible given privacy concerns. 

 --

 Are the conclusions drawn adequately supported by the results?

 --

 Given an inability to review full transcripts from the interviews, the results rely upon the summaries generated by the study authors. While there are two researchers which coded the results, there still could be some level of overlapping subjective interpretation. 

 --

 Summary

 --

 I am approving this manuscript, as it represents a large amount of work to organize these interviews. They give a good perspective on the Act and how it protects Zambian interests. In my comments, I have raised questions which could add additional perspective. However, some of these may not be addressable with the current survey answers, and it is beyond the scope to add additional or updated interviews.

 The manuscript does give an excellent perspective on attitudes towards genomic research in Zambia, and how the Act is structured to protect those interest. As such, it is an important perspective for those interested in performing genomic studies in collaboration with Zambian researchers.

Is the work clearly and accurately presented and does it cite the current literature?

Yes

If applicable, is the statistical analysis and its interpretation appropriate?

Not applicable

Are all the source data underlying the results available to ensure full reproducibility?

No source data required

Is the study design appropriate and is the work technically sound?

Yes

Are the conclusions drawn adequately supported by the results?

Partly

Are sufficient details of methods and analysis provided to allow replication by others?

Partly

Reviewer Expertise:

Genomic analyses, cohort studies, bioinformatics

I confirm that I have read this submission and believe that I have an appropriate level of expertise to confirm that it is of an acceptable scientific standard.

# Reviewer report: Reviewer response for version 2

**DOI:** 10.21956/aasopenres.16779.r31915

**Published:** 2024-12-04

**Authors:** Erisa Sabakaki Mwaka

**Affiliations:** 1 Department of Anatomy, School of Biomedical Sciences, Makerere University College of Health Sciences, Kampala, Uganda

I have no further comments to make. Approved.

Is the work clearly and accurately presented and does it cite the current literature?

Partly

If applicable, is the statistical analysis and its interpretation appropriate?

Not applicable

Are all the source data underlying the results available to ensure full reproducibility?

Yes

Is the study design appropriate and is the work technically sound?

Yes

Are the conclusions drawn adequately supported by the results?

Partly

Are sufficient details of methods and analysis provided to allow replication by others?

Yes

Reviewer Expertise:

ELSI of biobanking and genomic research, Research ethics, ELSI of using mobile phones for public health research, Human Anatomy, Orthopaedics and Bone health in HIV.

I confirm that I have read this submission and believe that I have an appropriate level of expertise to confirm that it is of an acceptable scientific standard.

# Reviewer report: Reviewer response for version 1

**DOI:** 10.21956/aasopenres.14191.r27904

**Published:** 2020-09-21

**Authors:** Erisa Sabakaki Mwaka

**Affiliations:** 1 Department of Anatomy, School of Biomedical Sciences, Makerere University College of Health Sciences, Kampala, Uganda

**General remarks**

 This is a very important study because it has come at a time when there is a push towards open data sharing and the sharing of data/samples with commercial entities. This article should interest many stakeholders on the African continent.

 I have had the chance to read through Reviewer 1’s comments and will not repeat them.

**Introduction**

 Whereas the study title indicates genomic research, most of the content pertains to biobanking (storage and future use of samples/data). Is genomic research and the storage of samples for future use synonymous? Please include a paragraph on the relationship between the two in the introduction or in the preamble to the results for the reader who might not be well versed with these terms.

**Results**

 Were the three themes derived from the data or were they pre-determined in the interview guide? Please clarify this in the manuscript.

**Methods**

 What is the importance of the brief biography on the first author in describing the methods? Was this aimed at stressing that OM was well conversant with the HRA? Shouldn’t this be transferred to an another appropriate section of the manuscript?

*“Specifically, in the interviews, we explored participants’ views on genomics research and broad consent in the context of health research and the history and capacity of the Zambian Health Research Act of 2013 to regulate genomics in Zambia”*. However, there is very limited information in the results on broad consent despite having several questions in the interview guide? Might it be that participants did not understand broad consent well? Please explain.

**R *esults***

*“Generally, the policymakers we interviewed acknowledged that the Act in its ‘current’ form was limited in its ability to provide adequate regulation of genomics research*”. Much as most participants seemed to support the stringency of the HRA, did they suggest ways of mitigating the potential detrimental consequences this law on the biomedical research industry in Zambia? If so, please add a paragraph on this (if possible).

 Some quotes should be edited to ensure smooth reading.

**Conclusion**

 From your results what recommendations do you have in regard to advancing genomics and biobanking in international collaborative research in Zambia?

Is the work clearly and accurately presented and does it cite the current literature?

Partly

If applicable, is the statistical analysis and its interpretation appropriate?

Not applicable

Are all the source data underlying the results available to ensure full reproducibility?

Yes

Is the study design appropriate and is the work technically sound?

Yes

Are the conclusions drawn adequately supported by the results?

Partly

Are sufficient details of methods and analysis provided to allow replication by others?

Yes

Reviewer Expertise:

ELSI of biobanking and genomic research, Research ethics, ELSI of using mobile phones for public health research, Human Anatomy, Orthopaedics and Bone health in HIV.

I confirm that I have read this submission and believe that I have an appropriate level of expertise to confirm that it is of an acceptable scientific standard, however I have significant reservations, as outlined above.

# Response

**Published:** 2024-09-20

**Authors:** Oliver Mweemba

**Affiliations:** University of Zambia, Zambia

General remarks:

**Comment: **This is a very interesting and important study examining the perspectives of key stakeholders about genomic research and specifically how the National Health Research Act impact this field of research. This study is important not only because of the global increase in genomic research and the interest in harnessing the diversity of Africa’s genetic heritage internationally, but also because Zambia has an especially restrictive policy which differs from many other countries on the continent. In this regard, relevant parts of the Act prohibit “future unspecified health research” and significantly restricts the “storage, export, and re-use of tissue samples”. Interestingly, while this restrictive policy could potentially isolate the country from future genomic research which could be of benefit to the Zambian population, participants appeared to be supportive of these provisions of the Act.  

**Response**: This is well noted with thanks    

**Comment**: It was very useful to have the extended data referenced in this paper and accessible for review (Extended data for the Genomics Study in Zambia.docx).  

**Response: **Noted with thanks

**comment:** The following statement is not clear: “While the H3Africa initiative aims to establish biobanks in Africa, much other genomics research, where the export of samples and data for use elsewhere, continues to be common, partly because of mandatory policies for the sharing of samples and data.” Which mandatory policies are referred to, especially regarding the sharing of samples? The comma should be removed to improve readability: “where the export of samples and data for use elsewhere, continues to be common”.

**Response**: While the H3Africa initiative aims to establish biobanks in Africa, much of the other genomics research, where the export of samples and data for use elsewhere, continues to be common, partly because of mandatory policies for the sharing of samples and data.” Which mandatory policies are referred to especially regarding the sharing of samples? see page 5. **Comment: **“A particular challenge in this regard is that the human capacity and infrastructure required to successfully conduct genomics research remains concentrated in particular African countries (Aron *et al*., 20171), with others relying on collaboration to make this happen (Yakubu *et al*., 20182).” The last reference seems incorrect since it deals with a “Model framework for governance of genomic research and biobanking in Africa” and does not, as far as I could ascertain, contain information about specific countries relying on collaboration. **Response**: The reference has been replaced by   Munung, N. S., Mayosi, B. M., & de Vries, J. (2017). Equity in international health research collaborations in Africa: Perceptions and expectations of African researchers. *PloS one*, *12*(10), e0186237. https://doi.org/10.1371/journal.pone.0186237. See introduction section  

**Comment: **“The first is a requirement that no biological materials may be collected for ‘future unspecified health research’ (HRA Section 47(1)) – effectively prohibiting the use of blanket, broad or tiered consent (although exactly what counts as ‘unspecified' could be debated).” It would be helpful for readers if the authors could provide more detail about which consent models, if any, are allowed in the HRA. It is also not clear how a restriction on “future unspecified health research” could preclude tiered consent since with this kind of consent, all the options can be specified. For instance, Nembaware et al, state the following: “Tiered informed consent addresses these challenges by providing detailed information about the intended specimen/data use and storage, thus enabling participants to individually select a level of specimen and/or data sharing through responses to specific questions” (Nembaware et al. A framework for tiered informed consent for health genomic research in Africa. October 2019. Nature Genetics 51(11)3. The authors state that “exactly what counts as ‘unspecified' could be debated”. Has no scholarship been done in this regard?  

**Response:** The HRA does not specifically allow any consent models.

**Comment: **The following statement should be rephrased to make it clear that the researchers, and not the participants, did the explaining: “All the participants provided written consent after explaining the purpose of the study”.

**Response: **The statement has been rephrased as All the participants provided written consent after the researchers explained the purpose of the study”. See page 8

**comment**: Had the interview guide been pilot tested?

**Response**: The interview guide had been pilot tested and modified after this process, as clarified in the methods section.  See section data collection section

**Comment: **Could the researchers please clarify which interview guide had been used for participants who were neither in a Research Ethics Committee nor a Research Regulator, for instance the two scientists involved in genomics research and the one community representative.  

**Response**: The same interview guide was used for all participants for this paper. The two scientist and community representative were members of the research ethics committee. See section on data collection

**Comment:** Which participants were regarded as Research Regulators?

**Response**: Participants regarded as regulators were those from institutional review boards and those who were involved in the development of the Act. See section on data collection **Comment: **Despite stated as a major focus of the interviews, it is not clear which questions explored the “capacity of the Zambian Health Research Act of 2013 to regulate genomics in Zambia”.  

**Response**: Thank you for your insightful comment. We apologize for any confusion regarding the specific questions that explored the capacity of the Zambian Health Research Act of 2013 to regulate genomics in Zambia. We used a semi-structured interview guide that included questions designed to elicit participants' perspectives on the Act's relevance, adequacy, and enforceability in the context of genomics research.

**Comment**: Were any questions asked about commercialisation and whether that would impact on participants’ view about exportation and sharing of samples? If not, this should be added as a limitation to the study, since this is such a fundamental concern in these debates. **Response**: Questions about commercialization where not asked. This has been added as a limitation of the study. See limitations section

**Comment: **“Primary amongst these was a worry about the potential for genomic resources to be misused in ways that offend the culture or religion of research participants, or that may be stigmatising or exploitative in nature.” This statement can be strengthened by including relevant examples of such exploitation, for instance Moodley & Kleinsmidt. Allegations of misuse of African DNA in the UK: Will data protection legislation in South Africa be sufficient to prevent arecurrence? Developing World Bioeth. 2020;00:1–64. And, of course, the Havasupai case: Mello MM, Wolf LE. The Havasupai Indian tribe case--lessons for research involving stored biologic samples. New England Journal of Medicine. 2010; 363(3):204–75.

**Response: **Examples on the potential misuse of genomics resources have been included. See discussion section  

**Comment: **It may also be worthwhile mention issues around the problem that the genetic diversity of African populations may make them uniquely re-identifiable by their genomic profiles and genome summary data, which is of specific concern if data have to be made “available openly on the internet without access oversight or controls”, as required by the new National Institutes of Health policy – see Tiffin, N. Potential risks and solutions for sharing genome summary data from African populations. BMC Med Genomics **12, **152 (2019)6. **Response:** issues around the problem that the genetic diversity of African populations may make them uniquely re-identifiable by their genomic profiles and genome summary data, which is of specific concern if data have to be made “available openly on the internet without access oversight or controls”, as required by the new National Institutes of Health policy have been included in the discussion section and the reference added. see page 16  

**Comment: **In addition, the researchers can highlight the problem of authorship and recognition of African researchers in the field of genomic research – see Adedokun, B O, Olopade, C O and Olopade, O I 2016 Building local capacity for genomics research in Africa: recommendations from analysis of publications in Sub-Saharan Africa from 2004 to 2013. *Global Health Action*, 9: 310267.  

**Response**: The problem of authorship and recognition of African researchers in the field of genomics research has been highlighted and the reference included. see discussion section. **Comment: **“Petti *et al. *(2006)8 suggest that performing laboratory tests locally or at least regionally gives greater African ownership of studies and high income countries collaborators need to help build research capacity.” The reference used does not appear to be appropriate since it deals with laboratory diagnostic capacity and not research. In addition, it does not address the issue of ownership at all.  

**Response**: The reference has been removed.  See page 15.  

**Comment:** The researchers could consider adding this article to the discussion since it highlights specific issues around the lack of computing infrastructure in the context of genomic research in Africa: Mulder, N et al. 2017 Genomic Research Data Generation, Analysis and Sharing – Challenges in the African Setting. Data Science Journal, 16: 49, pp. 1–159. **Response**: The article has been included. See discussion section.  

**Comment:** “A worry is that the strict regulatory environment in Zambia could mean that the country and its researchers are avoided in future health research collaborations, thus isolating the country from potentially beneficial health research. Interestingly, our participants did not seem to share this concern and generally supported this provision of the Act”. It is not clear that this conclusion is supported by the results presented, especially when considering the following statement: “Importantly, the participants acknowledged that the stringency of the Act may preclude Zambians from effectively engaging in genomics research”.  

**Response: **The statement has been revised accordingly. see page 15.

**Comment: **Many questions were asked about broad consent but the only reference to answers to these questions is that specific consent was preferred. Since this is such a pertinent issue, it would be very useful if the researchers could expand on these findings. For instance, what were the general and ethical reasons given by participants for not supporting broad consent for genomic research? What were the concerns based on? etc.

**Response: **Findings have been expanded to include reasons given for not supporting broad consent for genomic research.

**comment:** It is not clear from the quote that the meaning was that “Zambian researchers should be supported to participate in international genomics research collaborations”: “If we are to understand disease dynamics, sometimes we need to do those studies. If we are to develop treatments for certain diseases, we need to know how these drugs operate in the human body, what changes happen in the chromosomes and so on... For clinical practice, if I could relate to drugs that is what I understand better yes probably because that could help us now individualise the management of patients. So as a medical person I think we need to encourage genomics research. (Researcher and Research Ethics Committee member).” There is also no such question in the interview guide. Did the researchers reach this conclusion since this specific participant responded to a prompt about international collaboration?

**Response: **We did not strictly follow the interview for all questions, even though some questions were not in the guide, we made some further probes to get clarification on some topics. We have included a sentence in the methods sections. See page 8  

**Comment:** I don’t quite understand this argument: “Interestingly, participants framed informed consent as being one way of protecting against sample misuse: “There is a likelihood that things that the person has not consented to are going to be done and of course we have had this issue that has been discussed left right and centre of how people from the western world, for example, want to manipulate Africans by coming here and harvest samples and things like that. Many people are in a way duped so to say because they may be enticed with something and they give these samples (Institutional Lawyer).” According to my interpretation of the quote, participants had consented to some testing, since the participant states that “things the participants has not consented to” implying that they had consented to some things. Could the researchers please clarify to avoid a misunderstanding of this statement.  

**Response:** The statement has been clarified to reflect that the participant expressed a concern about the ethical implications of research suggesting that there is a risk of exploitation where individuals may provide samples without fully understanding the potential consequences or benefits. see page 10.

**Comment**: Since consent is one of the major issues the researchers focused on, it is important to understand how many of the participants, and which kinds of participants, agreed with this statement: “Because of some of the concerns raised, specific consent was insisted upon as a possible solution to these concerns”. The researchers should therefore consider presenting such important statements in a semi-quantified manner, for example, Half of the NHREC members and all of the Regulators said...  

**Response**: This would add more value to this paper; however, the qualitative nature of the study does not allow for quantification to me made, this has been noted as a limitation to the study.                      

**Comment: **As also stated above, it does not seem to me that this statement precludes tiered consent since, in that model, research is still specified: “Because of some of the concerns raised, specific consent was insisted upon as a possible solution to these concerns. Specific consent is defined as consent for a particular research question as opposed to for instance tiered or broad consent, which allow for the re-use of samples and data for other research questions also. We have to specify what exactly the individual is consenting to, what can be done on that particular specimen they are submitting or they are donating and specifically that they would like the specimen to be used for the particular condition the participant consented for (Former Research Ethics Committee and NHREC member).” See Nembaware N et al. A framework for tiered informed consent for health genomic research in Africa. Nature Genetics 20193. “Tiered informed consent addresses these challenges by providing detailed information about the intended specimen/data use and storage, thus enabling participants to individually select a level of specimen and/or data sharing through responses to specific questions.” This is also an important article which should be referenced.  

**Response: **The reference has been included as provided. See discussion section on page 15 **Comment: **“Many of the participants placed emphasis on the importance of control and ownership by Zambian researchers and regulatory institutions over samples and data, which could leave samples open to abuse by unscrupulous researchers.” It would seem that the researchers should add that abuse could happen when samples are sent overseas and control is lost/ surrendered, otherwise this sentence could be misinterpreted.   Response; The statement abuse could happen when samples are sent overseas and control is lost/ surrendered has been added. See page 11.  

**Comment: ** Could the researchers please explain what is meant by “create the data” in the following quotation: “they have the monopoly on the data and the only thing you can do is create the data which you can’t access”. Does this imply research fabrication? Response: The creation of data in this sentence implies research fabrication as explained. see page 11 **Comment: **Please rephrase for clarity: “Past experience is that it is difficult for local researchers to access samples and data from Zambia that is stored and distributed from other countries...”. It should be clear that data is stored outside of Zambia. What is meant by “distributed” in this context?  

**Response: **The sentence has been revised accordingly. See page 11  

**Comment**: The second sentence seems unnecessary since, if all 14 were “involved either with the development of the Act or are charged with its implementation”, then 8 of this group will of course also be: “Importantly, out of the 14 interviewees – all of whom were involved either with the development of the Act or are charged with its implementation as members of ethics committees – about eight participants were conversant with the law and the rest had general ideas about it. All the eight participants who were conversant with the law were involved either with the development of the Act or its implementation.”  

**Response**: The sentence has been removed.  

**Comment: **Could this quotation perhaps be annotated to clarify what is meant: “it recognised that there was no legal framework in which the ethics would be empowered to follow-up studies, hence the need formulate the Act.” For instance, how can ethics be empowered? Should there be a “to” between “need formulate”?  

**Response: **it recognised that there was no legal framework in which the ethics would be empowered to follow-up studies, hence the need to formulate the Act.” See page 12 **Comment: **One of the quotes states: “I think there's a statement which says that "specimens belong to the Government of the Republic of Zambia". If true, this has important implications. Since the participant was not certainly about this fact, have the researchers checked the National Health Research Act to ascertain whether it is true?  

**Response:** According to the Act specimens from the citizens belong to the government as the Act stipulates.  This has been verified finding discussed in the findings section. See discussion section on page 15

**Comment: **Please reconsider the placement of the commas in this quotation: “conditions that affect our people here that we feel that the solutions, could be partly found from genomic research but then given, the stringency of our regulations it was going to be very difficult for us as a country to participate in genomic research”.

**Response: **Quotations have been placed in commas. See results section  

**Comment**: The word “extraction” does not seem to be the here: “there are provisions in place that would ensure the long-term reversal of a trend of sample extraction without benefits to Zambian researchers or the general public”.  

**Response**: The statement has been revised and the word extraction removed. See results on page 13.  

**Comment: **For clarity, the researchers should clarify that the participants referred to were from research ethics committees: “Interestingly, participants described how they sought to navigate this prohibition by seeking to get some clarity – at a broad level – of what future research could be conducted.”  

**Response**: This has been clarified in the manuscript. see page 14  

**Comment**: The concluding sentence appears to be somewhat unsubstantiated, especially when read after the preceding paragraph: “Our study suggests that the legislation is arguably not the best way to guard against perceived and actual unfair research practices, especially in the field of health research that is constantly changing. Regulation that has the ability to adapt to these changes may be better suited for that purpose.” I suggest that the researchers preface this conclusion with a few sentences that build up to and support it.  

**Response**: The concluding sentence has been revised and sentences added to support this conclusion. see conclusion on page 16

**Comment**: Finally, some grammatical errors need to be corrected:

 “The restrictive nature of HRA...”

 “Some policy makers also worried the samples and data that comes...” “human capacity and infrastructure ... remains...” “A signed copy ... were kept...”

**Response**: Grammatical errors have been corrected “The restrictive nature of HRA...” “Some policy makers also worried that the samples and data that comes...” “human capacity and infrastructure ... remains...”

 “A signed copy ... was kept...” See truck changes

# Reviewer report: Reviewer response for version 1

**DOI:** 10.21956/aasopenres.14191.r27759

**Published:** 2020-09-04

**Authors:** Theresa M. Rossouw

**Affiliations:** 1 Department of Immunology, School of Medicine, Faculty of Health Sciences, University of Pretoria, Pretoria, South Africa

General remarks:

 This is a very interesting and important study examining the perspectives of key stakeholders about genomic research and specifically how the National Health Research Act impact this field of research. This study is important not only because of the global increase in genomic research and the interest in harnessing the diversity of Africa’s genetic heritage internationally, but also because Zambia has an especially restrictive policy which differs from many other countries on the continent. In this regard, relevant parts of the Act prohibit “future unspecified health research” and significantly restricts the “storage, export, and re-use of tissue samples”. Interestingly, while this restrictive policy could potentially isolate the country from future genomic research which could be of benefit to the Zambian population, participants appeared to be supportive of these provisions of the Act.

 It was very useful to have the extended data referenced in this paper and accessible for review (Extended data for the Genomics Study in Zambia.docx).

 I fully support the researchers stance on restrictions placed on data availability for this study.

 My general opinion is that the article will be of interest to African and international scholars in the field of genomic research, that it will add to the existing body of literature in this field, and is therefore worthy of publication. I do, however, recommend that a few aspects be clarified, the methodology further expanded, and the references corrected, expanded and updated. I give specific recommendations in the sections below.

Introduction:

 The following statement is not clear: “While the H3Africa initiative aims to establish biobanks in Africa, much other genomics research, where the export of samples and data for use elsewhere, continues to be common, partly because of mandatory policies for the sharing of samples and data.” Which mandatory policies are referred to, especially regarding the sharing of samples? The comma should be removed to improve readability: “where the export of samples and data for use elsewhere, continues to be common”.

 “A particular challenge in this regard is that the human capacity and infrastructure required to successfully conduct genomics research remains concentrated in particular African countries (Aron *et al*., 2017 ^1^), with others relying on collaboration to make this happen (Yakubu *et al*., 2018 ^2^).” The last reference seems incorrect since it deals with a “Model framework for governance of genomic research and biobanking in Africa” and does not, as far as I could ascertain, contain information about specific countries relying on collaboration.

 “The first is a requirement that no biological materials may be collected for ‘future unspecified health research’ (HRA Section 47(1)) – effectively prohibiting the use of blanket, broad or tiered consent (although exactly what counts as ‘unspecified' could be debated).” It would be helpful for readers if the authors could provide more detail about which consent models, if any, are allowed in the HRA. It is also not clear how a restriction on “future unspecified health research” could preclude tiered consent since with this kind of consent, all the options can be specified. For instance, Nembaware et al, state the following: “Tiered informed consent addresses these challenges by providing detailed information about the intended specimen/data use and storage, thus enabling participants to individually select a level of specimen and/or data sharing through responses to specific questions” (Nembaware et al. A framework for tiered informed consent for health genomic research in Africa. October 2019. Nature Genetics 51(11) ^3^.

 The authors state that “exactly what counts as ‘unspecified' could be debated”. Has no scholarship been done in this regard?

 The following statement should be rephrased to make it clear that the researchers, and not the participants, did the explaining: “All the participants provided written consent after explaining the purpose of the study”.

Methodology:

 Had the interview guide been pilot tested?

 Could the researchers please clarify which interview guide had been used for participants who were neither in a Research Ethics Committee nor a Research Regulator, for instance the two scientists involved in genomics research and the one community representative.

 Which participants were regarded as Research Regulators?

 Despite stated as a major focus of the interviews, it is not clear which questions explored the “capacity of the Zambian Health Research Act of 2013 to regulate genomics in Zambia”.

 Were any questions asked about commercialisation and whether that would impact on participants’ view about exportation and sharing of samples? If not, this should be added as a limitation to the study, since this is such a fundamental concern in these debates.

Results:

 Many questions were asked about broad consent but the only reference to answers to these questions is that specific consent was preferred. Since this is such a pertinent issue, it would be very useful if the researchers could expand on these findings. For instance, what were the general and ethical reasons given by participants for not supporting broad consent for genomic research? What were the concerns based on? etc.

 It is not clear from the quote that the meaning was that “Zambian researchers should be supported to participate in international genomics research collaborations”: “If we are to understand disease dynamics, sometimes we need to do those studies. If we are to develop treatments for certain diseases, we need to know how these drugs operate in the human body, what changes happen in the chromosomes and so on… For clinical practice, if I could relate to drugs that is what I understand better yes probably because that could help us now individualise the management of patients. So as a medical person I think we need to encourage genomics research. (Researcher and Research Ethics Committee member).” There is also no such question in the interview guide. Did the researchers reach this conclusion since this specific participant responded to a prompt about international collaboration?

 I don’t quite understand this argument: “Interestingly, participants framed informed consent as being one way of protecting against sample misuse: “There is a likelihood that things that the person has not consented to are going to be done and of course we have had this issue that has been discussed left right and centre of how people from the western world, for example, want to manipulate Africans by coming here and harvest samples and things like that. Many people are in a way duped so to say because they may be enticed with something and they give these samples (Institutional Lawyer).” According to my interpretation of the quote, participants had consented to some testing, since the participant states that “things the participants has not consented to” implying that they had consented to some things. Could the researchers please clarify to avoid a misunderstanding of this statement.

 Since consent is one of the major issues the researchers focused on, it is important to understand how many of the participants, and which kinds of participants, agreed with this statement: “Because of some of the concerns raised, specific consent was insisted upon as a possible solution to these concerns”. The researchers should therefore consider presenting such important statements in a semi-quantified manner, for example, Half of the NHREC members and all of the Regulators said…

 As also stated above, it does not seem to me that this statement precludes tiered consent since, in that model, research is still specified: “Because of some of the concerns raised, specific consent was insisted upon as a possible solution to these concerns. Specific consent is defined as consent for a particular research question as opposed to for instance tiered or broad consent, which allow for the re-use of samples and data for other research questions also. We have to specify what exactly the individual is consenting to, what can be done on that particular specimen they are submitting or they are donating and specifically that they would like the specimen to be used for the particular condition the participant consented for (Former Research Ethics Committee and NHREC member).” See Nembaware N et al. A framework for tiered informed consent for health genomic research in Africa. Nature Genetics 2019 ^3^. “Tiered informed consent addresses these challenges by providing detailed information about the intended specimen/data use and storage, thus enabling participants to individually select a level of specimen and/or data sharing through responses to specific questions.” This is also an important article which should be referenced.

 “Many of the participants placed emphasis on the importance of control and ownership by Zambian researchers and regulatory institutions over samples and data, which could leave samples open to abuse by unscrupulous researchers.” It would seem that the researchers should add that abuse could happen when samples are sent overseas and control is lost/ surrendered, otherwise this sentence could be misinterpreted.

 Could the researchers please explain what is meant by “create the data” in the following quotation: “they have the monopoly on the data and the only thing you can do is create the data which you can’t access”. Does this imply research fabrication?

 Please rephrase for clarity: “Past experience is that it is difficult for local researchers to access samples and data from Zambia that is stored and distributed from other countries...”. It should be clear that data is stored outside of Zambia. What is meant by “distributed” in this context?

 The second sentence seems unnecessary since, if all 14 were “involved either with the development of the Act or are charged with its implementation”, then 8 of this group will of course also be: “Importantly, out of the 14 interviewees – all of whom were involved either with the development of the Act or are charged with its implementation as members of ethics committees – about eight participants were conversant with the law and the rest had general ideas about it. All the eight participants who were conversant with the law were involved either with the development of the Act or its implementation.”

 Could this quotation perhaps be annotated to clarify what is meant: “it recognised that there was no legal framework in which the ethics would be empowered to follow-up studies, hence the need formulate the Act.” For instance, how can ethics be empowered? Should there be a “to” between “need formulate”? 

 One of the quotes states: “I think there's a statement which says that "specimens belong to the Government of the Republic of Zambia". If true, this has important implications. Since the participant was not certainly about this fact, have the researchers checked the National Health Research Act to ascertain whether it is true?

 Please reconsider the placement of the commas in this quotation: “conditions that affect our people here that we feel that the solutions, could be partly found from genomic research but then given, the stringency of our regulations it was going to be very difficult for us as a country to participate in genomic research”.

 The word “extraction” does not seem to be the here: “there are provisions in place that would ensure the long-term reversal of a trend of sample extraction without benefits to Zambian researchers or the general public”.

 For clarity, the researchers should clarify that the participants referred to were from research ethics committees: “Interestingly, participants described how they sought to navigate this prohibition by seeking to get some clarity – at a broad level – of what future research could be conducted.”

Discussion:

 “Primary amongst these was a worry about the potential for genomic resources to be misused in ways that offend the culture or religion of research participants, or that may be stigmatising or exploitative in nature.” This statement can be strengthened by including relevant examples of such exploitation, for instance Moodley & Kleinsmidt. Allegations of misuse of African DNA in the UK: Will data protection legislation in South Africa be sufficient to prevent a

 recurrence? Developing World Bioeth. 2020;00:1–6 ^4^. And, of course, the Havasupai case: Mello MM, Wolf LE. The Havasupai Indian tribe case--lessons for research involving stored biologic samples. New England Journal of Medicine. 2010; 363(3):204–7 ^5^.

 It may also be worthwhile mention issues around the problem that the genetic diversity of African populations may make them uniquely re-identifiable by their genomic profiles and genome summary data, which is of specific concern if data have to be made “available openly on the internet without access oversight or controls”, as required by the new National Institutes of Health policy – see Tiffin, N. Potential risks and solutions for sharing genome summary data from African populations. BMC Med Genomics **12, **152 (2019) ^6^.

 In addition, the researchers can highlight the problem of authorship and recognition of African researchers in the field of genomic research – see Adedokun, B O, Olopade, C O and Olopade, O I 2016 Building local capacity for genomics research in Africa: recommendations from analysis of publications in Sub-Saharan Africa from 2004 to 2013. *Global Health Action*, 9: 31026 ^7^.

 “Petti *et al.* (2006) ^8^ suggest that performing laboratory tests locally or at least regionally gives greater African ownership of studies and high income countries collaborators need to help build research capacity.” The reference used does not appear to be appropriate since it deals with laboratory diagnostic capacity and not research. In addition, it does not address the issue of ownership at all.

 The researchers could consider adding this article to the discussion since it highlights specific issues around the lack of computing infrastructure in the context of genomic research in Africa: Mulder, N et al. 2017 Genomic Research Data Generation, Analysis and Sharing – Challenges in the African Setting. Data Science Journal, 16: 49, pp. 1–15 ^9^.

 “A worry is that the strict regulatory environment in Zambia could mean that the country and its researchers are avoided in future health research collaborations, thus isolating the country from potentially beneficial health research. Interestingly, our participants did not seem to share this concern and generally supported this provision of the Act”. It is not clear that this conclusion is supported by the results presented, especially when considering the following statement: “Importantly, the participants acknowledged that the stringency of the Act may preclude Zambians from effectively engaging in genomics research”.

 The concluding sentence appears to be somewhat unsubstantiated, especially when read after the preceding paragraph: “Our study suggests that the legislation is arguably not the best way to guard against perceived and actual unfair research practices, especially in the field of health research that is constantly changing. Regulation that has the ability to adapt to these changes may be better suited for that purpose.” I suggest that the researchers preface this conclusion with a few sentences that build up to and support it.   

 Finally, some grammatical errors need to be corrected:

 “The restrictive nature of HRA…”

 “Some policy makers also worried the samples and data that comes…”

 “human capacity and infrastructure … remains…”

 “A signed copy … were kept…”

Is the work clearly and accurately presented and does it cite the current literature?

Partly

If applicable, is the statistical analysis and its interpretation appropriate?

Yes

Are all the source data underlying the results available to ensure full reproducibility?

Yes

Is the study design appropriate and is the work technically sound?

Yes

Are the conclusions drawn adequately supported by the results?

Partly

Are sufficient details of methods and analysis provided to allow replication by others?

Partly

Reviewer Expertise:

HIV immunology and drug resistance; Biomedical and research ethics

I confirm that I have read this submission and believe that I have an appropriate level of expertise to confirm that it is of an acceptable scientific standard, however I have significant reservations, as outlined above.

## References

[1] : H3ABioNet: Developing Sustainable Bioinformatics Capacity in Africa. *EMBnet.journal* .2017;23(0) : 10.14806/ej.23.0.886 10.14806/ej.23.0.886

[2] : Model framework for governance of genomic research and biobanking in Africa - a content description. *AAS Open Res* .2018;1: 10.12688/aasopenres.12844.2 13 10.12688/aasopenres.12844.2 30714023 PMC6354903

[3] : A framework for tiered informed consent for health genomic research in Africa. *Nat Genet* .51(11) : 10.1038/s41588-019-0520-x 1566-1571 10.1038/s41588-019-0520-x 31659323 PMC7258308

[4] : Allegations of misuse of African DNA in the UK: Will data protection legislation in South Africa be sufficient to prevent a recurrence?. *Dev World Bioeth* .2020; 10.1111/dewb.12277 10.1111/dewb.12277 32767549

[5] : The Havasupai Indian tribe case--lessons for research involving stored biologic samples. *N Engl J Med* .2010;363(3) : 10.1056/NEJMp1005203 204-7 10.1056/NEJMp1005203 20538622

[6] : Potential risks and solutions for sharing genome summary data from African populations. *BMC Medical Genomics* .2019;12(1) : 10.1186/s12920-019-0604-6 10.1186/s12920-019-0604-6 31684976 PMC6830006

[7] : Building local capacity for genomics research in Africa: recommendations from analysis of publications in Sub-Saharan Africa from 2004 to 2013. *Glob Health Action* .2016;9: 10.3402/gha.v9.31026 31026 10.3402/gha.v9.31026 27178644 PMC4867048

[8] : Laboratory medicine in Africa: a barrier to effective health care. *Clin Infect Dis* .2006;42(3) : 10.1086/499363 377-82 10.1086/499363 16392084

[9] : Genomic Research Data Generation, Analysis and Sharing – Challenges in the African Setting. *Data Science Journal* .2017;16: 10.5334/dsj-2017-049 10.5334/dsj-2017-049

# Response

**Published:** 2024-09-20

**Authors:** Oliver Mweemba

**Affiliations:** University of Zambia, Zambia

**Comment: **This is a very important study because it has come at a time when there is a push towards open data sharing and the sharing of data/samples with commercial entities. This article should interest many stakeholders on the African continent.

**Response**: This is well noted with thanks

**I**

**ntroduction** **Comment: **Whereas the study title indicates genomic research, most of the content pertains to biobanking (storage and future use of samples/data). Is genomic research and the storage of samples for future use synonymous? Please include a paragraph on the relationship between the two in the introduction or in the preamble to the results for the reader who might not be well versed with these terms.

**Response**: A paragraph has been added on the relationship between genomics research and biobanking in the introduction section. See introduction section, paragraph 3.

**Results** **Comment: **Were the three themes derived from the data or were they pre-determined in the interview guide? Please clarify this in the manuscript.  

**Response: **The three major themes were derived from the data. This has been clarified in the manuscript. See paragraph under data analysis

**Methods** **Comment: **What is the importance of the brief biography on the first author in describing the methods? Was this aimed at stressing that OM was well conversant with the HRA? Shouldn’t this be transferred to another appropriate section of the manuscript? **Response:** The biography of OM has been moved to the section on about the author. Section on About the author

**Comment: **“Specifically, in the interviews, we explored participants’ views on genomics research and broad consent in the context of health research and the history and capacity of the Zambian Health Research Act of 2013 to regulate genomics in Zambia”. However, there is very limited information in the results on broad consent despite having several questions in the interview guide? Might it be that participants did not understand broad consent well? Please explain.

**Response**: The reviewer's observation regarding the limited discussion of broad consent is valid. While we did indeed explore participants' views on broad consent in our interviews, we acknowledge that our analysis may not have adequately captured the nuances of their responses on this topic.

**R *esults*** **Comment:** *“Generally, the policymakers we interviewed acknowledged that the Act in its ‘current’ form was limited in its ability to provide adequate regulation of genomics research*”. Much as most participants seemed to support the stringency of the HRA, did they suggest ways of mitigating the potential detrimental consequences this law on the biomedical research industry in Zambia? If so, please add a paragraph on this (if possible). Some quotes should be edited to ensure smooth reading.

**Response: **The quotes have been edited to ensure smooth reading.

**Conclusion** **Comment: **From your results what recommendations do you have in regard to advancing genomics and biobanking in international collaborative research in Zambia? **Response: **From the study findings, we recommend the formation of regional biobanks that can be used to store samples from African countries giving access to researchers who would like to perform different tests on stored samples. See recommendations section.
